# A Vision-Based Deep Learning Framework for Monitoring and Recognition of Chemical Laboratory Operations

**DOI:** 10.3390/s26041106

**Published:** 2026-02-08

**Authors:** Chuntao Guo, Jing Lin, Shunxing Bao, Xin Liu, Yaru Wang, Yunlin Chen

**Affiliations:** 1 School of Physical Science and Engineering, Beijing Jiaotong University, Beijing 100044, China; 2 Beijing Purkinje General Instrument Co., Ltd., Beijing 100081, China; 3 Nanjing Institute of Measurement and Testing Technology, Nanjing 210049, China; 4 Institute of Analysis and Testing, Beijing Academy of Science and Technology (Beijing Center for Physical and Chemical Analysis), Beijing 100089, China; 5 School of Bioengineering, Beijing Polytechnic University, Beijing 100021, China

**Keywords:** vision-based sensing, pipetting behavior, pose estimation, spatiotemporal modeling, laboratory quality assurance

## Abstract

Standardized operating procedures are essential for ensuring safety and reproducibility in chemical laboratory experiments. However, real-time monitoring of manual laboratory operations, such as pipetting, remains challenging due to complex human–tool interactions, temporal dependencies between procedural steps, and operator variability. In this study, we propose a vision-based deep learning framework that leverages spatiotemporal features for automated monitoring of pipetting operations using non-contact visual sensing. Briefly, human poses and pipette interactions are extracted from video recordings using a YOLO-based perception model, while temporal execution patterns are captured through bidirectional long short-term memory networks. Experimental results demonstrate that the proposed approach can reliably distinguish between standard and non-standard pipetting behaviors across multiple predefined error categories and shows improved robustness compared with static or frame-level analysis. Overall, this work demonstrates the feasibility of vision-based AI systems for objective and scalable monitoring of laboratory pipetting operations, with potential applicability to other manual laboratory procedures.

## 1. Introduction

Chemical laboratory experiments rely on standardized operating procedures to ensure experimental safety, result reliability, and procedural reproducibility [1,2]. Deviations from prescribed operation steps—such as improper tool handling or incorrect execution order—can lead to measurement errors, safety risks, and inefficient use of laboratory resources [3,4]. In practice, supervision of laboratory operations is still largely dependent on manual observation and post hoc evaluation by supervisors [5]. Such approaches are labor-intensive and difficult to scale, particularly in training environments or laboratories with high experimental throughput [5]. Consequently, there is a pressing need for objective and automated methods capable of monitoring human operational activities in laboratory settings [6,7].

**Figure 1:**
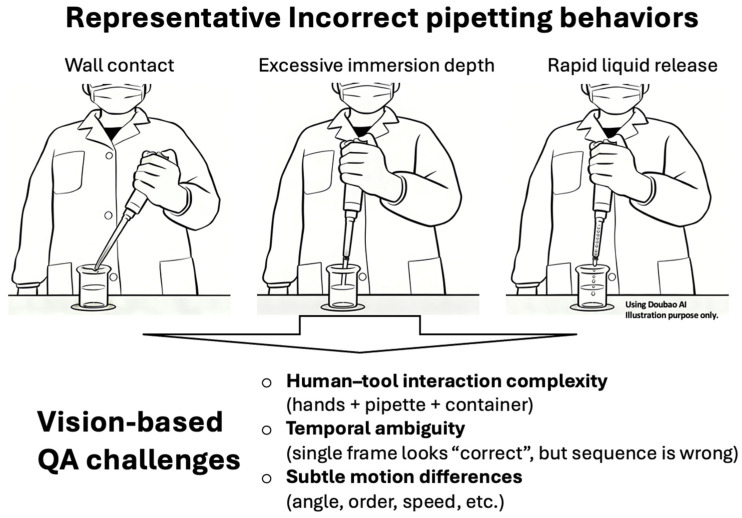
Representative incorrect pipetting behaviors and key challenges for vision-based QA in chemical laboratory environments.

Recent advances in vision-based sensing and artificial intelligence have enabled non-contact monitoring of human actions through camera systems, offering a promising alternative to traditional supervision methods [8,9]. Compared with wearable or instrumented sensors, vision-based approaches can capture rich spatial information without interfering with experimental procedures [9]. However, accurately recognizing laboratory operations remains challenging due to complex human–tool interactions and strong temporal dependencies between procedural steps [10,11]. Many existing approaches rely on frame-level detection or static posture analysis, which limits their ability to capture sequential dependencies and subtle operational errors [12]. These challenges motivate the development of integrated vision-based sensing frameworks that jointly model spatial and temporal information for reliable operation monitoring [13,14]. Related spatiotemporal and context-aware modeling approaches have been explored in other safety-critical domains, such as flight arrival time prediction and air traffic control communication analysis [15,16]. Although these studies address different applications, they demonstrate the broader value of integrating temporal modeling with domain-specific constraints for standardized operation monitoring. In chemical laboratory environments, similar challenges arise due to complex human–tool interactions and strict procedural requirements. Figure 1 illustrates common incorrect pipetting behaviors and the associated quality assurance (QA) challenges for vision-based monitoring, including complex human–tool interactions, temporal ambiguities and subtle motion difference.

To address these challenges, this study develops an integrated vision-based sensing framework that combines spatial perception of human actions with temporal modeling of operation sequences. Human pose information and tool interactions are extracted from video streams using a YOLO-based pose estimation model, enabling robust detection of key body joints and relevant objects under varying experimental conditions [17,18]. Temporal dependencies between consecutive procedural steps are modeled using a bidirectional long short-term memory (BiLSTM) network, which captures the evolution of pose and motion features over time [19,20]. This spatiotemporal representation allows the system to distinguish subtle differences between correct and incorrect operations that may not be identifiable from static frames alone [21].

Based on the extracted spatiotemporal features, multiple classification models are evaluated to assess operational correctness across predefined laboratory error categories [22]. Rather than introducing new learning architectures, this work focuses on system integration and makes novel contributions by formulating laboratory pipetting quality assessment as a sequence-level vision problem with explicit, standard-driven error definitions, and by integrating pose-based human–tool perception with temporal execution modeling to capture subtle procedural deviations beyond frame-level analysis. The proposed framework operates as a non-contact, camera-based sensing system that can be deployed without modifying existing laboratory instruments or experimental workflows, making it suitable for routine laboratory training settings [23]. The main contributions of this study are threefold:

- Development of a vision-based sensing framework for automated monitoring of laboratory operations.
- Integration of spatial pose perception with temporal sequence modeling to enhance recognition robustness.
- Experimental validation across multiple operational error categories, demonstrating feasibility for objective laboratory monitoring.

## 2. Materials and Methods

### 2.1. Problem Definition and System Overview

In this study, improper pipetting behaviors are defined as observable deviations from predefined procedural criteria in chemical laboratory workflows. The problem is formulated as automated monitoring of pipetting operations using non-contact vision-based sensing. Given a video sequence capturing a complete pipetting process, the objective is to determine whether the operation conforms to procedural criteria and, when applicable, to identify specific categories of incorrect behaviors.

To address this task, an integrated vision-based sensing framework is proposed that combines spatial perception of human pose and tool interactions with temporal modeling of operation sequences. Video streams captured by a fixed camera are processed to extract pose- and motion-related features, which are modeled over time to enable classification of correct and incorrect operations. The incorrect pipetting behavior categories considered in this study are defined using objective operational criteria, as summarized in Table 1. The operational definitions are based on established laboratory operating standards and good laboratory practice guidelines, including relevant Chinese national standards (e.g., GB/T 27407-2010 [24] and GB/T 27476.5-2014 [25]) and internationally adopted GLP/GMP regulations. Detailed threshold definitions and numerical values for each error category are provided in the Appendix A (Table A1). These thresholds are derived from standard laboratory operating procedures and expert practice to ensure interpretability and procedural relevance. Each video sequence corresponds to a complete pipetting operation and is treated as one analysis sample.

### 2.2. Overall Framework Workflow

**Figure 2:**
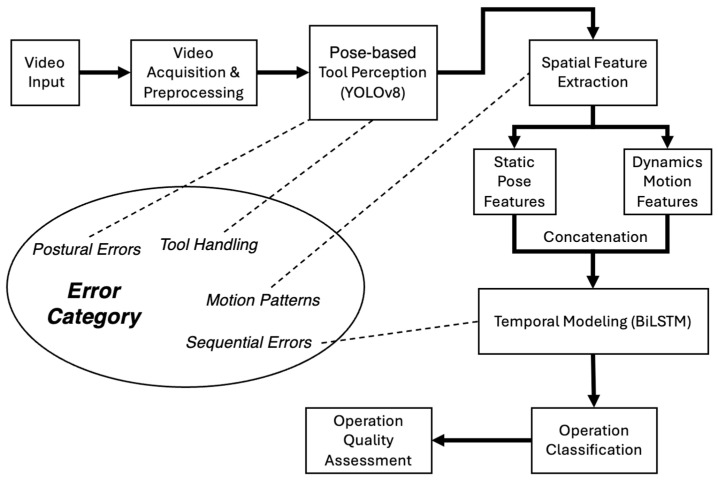
Overall workflow of the proposed vision-based sensing framework for chemical laboratory operation monitoring. The defined error categories correspond to different spatial and temporal characteristics captured by the framework.

Figure 2 illustrates the overall workflow of the proposed vision-based sensing framework for automated monitoring of pipetting operations. The framework follows a modular design consisting of spatial perception, feature representation, temporal modeling, and classification.

Video sequences capturing laboratory operations are first processed to extract spatial information related to human pose and tool configuration using a YOLOv8-based pose estimation module. From these spatial observations, two complementary feature representations are constructed: a static stream that captures pose configurations at individual time points, and a motion stream that captures temporal changes in pose and tool movement across consecutive frames based on inter-frame feature differences. These two streams encode complementary spatial and dynamic characteristics of pipetting behaviors.

The static and motion features are concatenated to form a unified spatiotemporal representation, which is subsequently modeled using a recurrent temporal network to capture execution order and temporal dependencies across the operation sequence. Finally, the learned sequence-level features are passed to a classification stage to determine operational correctness and identify predefined categories of incorrect pipetting behaviors.

### 2.3. Vision-Based Pose and Tool Perception

Vision-based pose and tool perception is employed to extract spatial observations relevant to pipetting operations from monocular video streams. Human body keypoints and pipette-related geometric features are detected using a YOLOv8-based pose estimation model [26], which provides efficient and accurate localization of skeletal joints and tool landmarks in real time. YOLOv8 is adopted due to its favorable trade-off between detection accuracy and computational efficiency, making it suitable for laboratory monitoring scenarios. From each video frame, the model outputs 2D keypoint coordinates of the upper body and hands, along with bounding boxes and orientation cues associated with the pipette. These spatial observations are organized as time-ordered pose vectors and serve as the input to subsequent feature extraction and temporal modeling stages.

### 2.4. Multimodal Feature Extraction

To characterize both instantaneous posture and temporal motion patterns during pipetting, a dual-stream feature extraction strategy is adopted, consisting of a static pose stream and a motion stream. Both features are concatenated to form a unified spatiotemporal representation that is further passed to the temporal modeling module for sequence-level analysis (Section 2.5).

#### 2.4.1. Static Pose Stream

The static pose stream encodes skeletal joint positions at each time step [27]. Let

(1) $$p\left(t\right)=\left[x_{1}\left(t\right),y_{1}\left(t\right),\ldots ,x_{J}\left(t\right),y_{J}\left(t\right)\right]$$

denote the 2D coordinates of J detected joints at time t. To capture local kinematic information, first- and second-order temporal derivatives are computed:

(2) ∆p(t)=p(t)−p(t−∆t)

(3) $$v(t)=\frac{∆p(t)}{∆t}$$

(4) $$a(t)=\frac{v(t)-v(t-∆t)}{∆t}$$

where these features encode joint displacement (Equation (2)), velocity (Equation (3)), and acceleration (Equation (4)), capturing posture stability and fine-grained motion cues relevant to operational correctness.

#### 2.4.2. Dynamics Motion Stream

The dynamic motion stream captures local appearance changes associated with hand and tool movements during pipetting. Motion features are extracted from pose-guided regions of interest to emphasize relevant hand–pipette interactions while reducing background interference. A motion-enhanced Weber Local Descriptor (WLD) is employed to encode relative intensity variations and directional changes, providing a compact representation of motion dynamics that is robust to illumination variation [28]. The resulting motion features reflect execution characteristics such as smoothness and abrupt movement changes. The extracted motion features encode execution characteristics, including movement continuity and abrupt transitions.

### 2.5. Temporal Modeling of Operation Sequences

Pipetting operations consist of temporally ordered actions that cannot be reliably interpreted from individual frames. To capture sequential dependencies and execution order, the extracted spatial and motion features are modeled over time using a bidirectional long short-term memory (BiLSTM) network. By processing feature sequences in both forward and backward directions, the BiLSTM captures contextual information from the entire operation, enabling discrimination between correct and incorrect execution patterns. The resulting sequence-level representations are subsequently used for operation-level classification.

### 2.6. Classification Models

Following spatiotemporal feature extraction, a classification stage is employed to assign each operation sequence to a predefined operational category. While temporal modeling captures motion dynamics and execution order, explicit classification is required to enable objective decision making and quantitative evaluation of operational correctness. To ensure that system performance is not dependent on a specific classifier and to support objective evaluation of the extracted spatiotemporal representations, seven representative classifiers are evaluated given the limited sample size. These include ID3, C4.5, AdaBoost, Naive Bayes, Bayesian Network, Random Forest, and Support Vector Machine (SVM). The selected classifiers cover diverse learning paradigms, allowing systematic assessment of classification performance across different decision mechanisms [29].

### 2.7. Use of Generative AI

Generative AI (ChatGPT 5.2) was used to assist with manuscript writing, including grammar correction and figure generation (Doubao-Seed-1.8, Schematic 1 only, for conceptual illustrations). All scientific analysis and conclusions were based on original research and expert judgment.

## 3. Experimental Setup

### 3.1. Dataset Collection and Annotation

Twelve participants were recruited for this study (Table 2), including six novice operators and six experienced operators. Each trial corresponds to one complete pipetting operation sequence and is treated as an independent sample for analysis. In total, the dataset consists of 48 operation sequences, including 24 erroneous (non-standard) sequences and 24 standard sequences. Each participant performed multiple pipetting trials following standardized laboratory protocols. Each trial corresponds to a complete pipetting operation and is treated as a single sequence-level sample for subsequent analysis. Operational correctness was manually assessed by a senior laboratory expert, with erroneous behaviors categorized according to the definitions summarized in Table 1. These sequence-level annotations serve as ground truth labels for error classification and evaluation.

In addition to sequence-level labels, frame-level annotations were generated to support visual modeling of procedural behavior. From the recorded laboratory videos, 15,000 frames were sampled across both correct and erroneous operations. Each frame was annotated with bounding boxes for two categories:

- anatomical joints used to characterize operator posture, and
- key laboratory apparatus, including pipettes, beakers, and test tubes.

This process produced approximately 5000 object-level annotations, which were used to train a YOLOv8-based object detector. Furthermore, 2500 frame sequences corresponding to predefined error types were separately identified and labeled to train and benchmark the error classification module.

### 3.2. Video Acquisition and Preprocessing

Visual data acquisition was performed using an Intel RealSense D455 stereo depth camera (Intel Corporation, Santa Clara, CA, USA), capturing synchronized RGB and depth streams at 30 Hz with a resolution of 1280 × 720. All pipetting operations were recorded using a fixed monocular camera positioned to capture the experimental workspace and operator hand movements. Video sequences were segmented into individual pipetting actions to ensure that each sample corresponded to a complete operation. Basic preprocessing steps, including frame resizing and temporal alignment, were applied to reduce variability across recordings and ensure consistent input for subsequent feature extraction and temporal modeling.

### 3.3. Training and Testing Protocol

Due to the limited sample size and to prevent data leakage, a participant-grouped cross-validation protocol was adopted. All sequences from the same participant were assigned to the same fold, ensuring that no participant appeared in both training and test sets. The 12 participants were randomly grouped into five folds (with 2–3 participants per fold), resulting in test set sizes of 8–12 sequences per fold (each participant contributing four sequences). Performance was reported as the average across the five folds.

### 3.4. Ablation Study Design

To assess the contribution of different feature components, a quantitative ablation study was conducted by selectively removing static pose features or motion features from the proposed framework. Three configurations were evaluated: static-only, motion-only, and combined static–motion features.

When both static and motion features were retained, temporal execution patterns were preserved and modeled using an LSTM-based temporal encoder followed by a fully connected classification layer. When either feature stream was removed, temporal structure was no longer fully available; therefore, non-temporal classifiers (SVM or Random Forest) were used, with the best-performing classifier reported for each reduced-feature configuration.

### 3.5. Evaluation Metrics

System performance was evaluated using standard classification metrics, including accuracy, precision, recall, and F1-score. Accuracy measures the proportion of correctly classified operation sequences across all categories. Precision and recall quantify the reliability of predicted incorrect behaviors and the system’s ability to detect true operational errors, respectively. The F1-score provides a balanced metric that integrates both precision and recall, particularly under limited or potentially imbalanced data conditions [30].

### 3.6. Computation Hardware Configuration and Software Implementation

All experiments and analyses were performed on a workstation equipped with an Intel Core i7-13700K CPU, 32 GB of DDR5 RAM, and an NVIDIA GeForce RTX 4090D GPU with 24 GB of GDDR6X memory. The software pipeline was implemented in Python 3.9, using PyTorch 2.0.1 for deep learning model development, TensorFlow 2.12 for auxiliary network training, and OpenCV 4.8.0 for real-time image processing and system integration.

## 4. Results

### 4.1. Action Reconstruction and Feature Analysis

**Figure 3:**
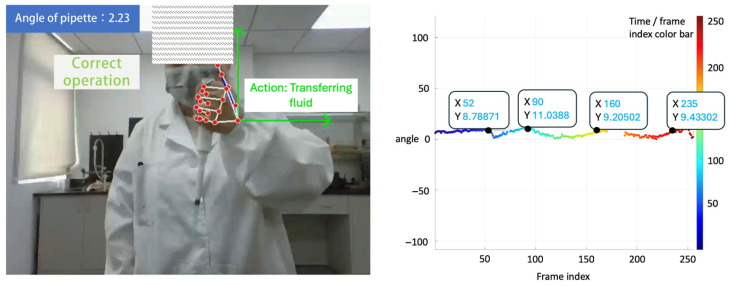
Hand action recognition and pose-based feature visualization: the pipette inclination angle is derived from pose-based keypoints, and color denotes temporal progression of the operation.

**Figure 4:**
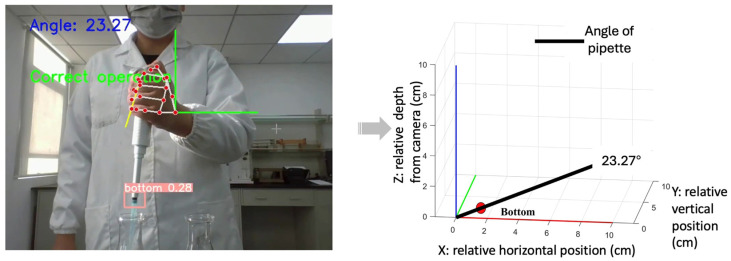
Pipette identification and orientation estimation Pose-based keypoints are used to estimate pipette orientation in a camera-centered 3D space, where X and Y denote image-plane directions and Z indicates relative depth. The pipette angle is derived from the orientation vector for posture assessment.

**Figure 5:**
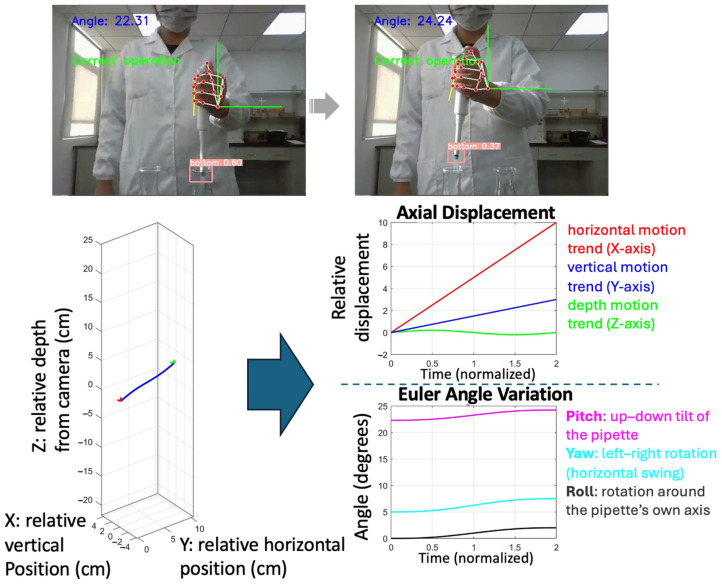
Hand and pipette trajectory recognition: Visualization of pipette motion and orientation during a representative operation. Relative 3D trajectory, axial displacement, and Euler angle variations illustrate coordinated positional and rotational dynamics over time.

**Figure 6:**
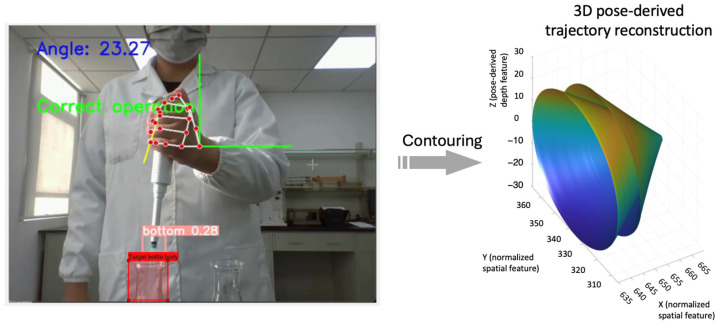
Position recognition of pipette and bottle body: Visualization of pose-derived spatiotemporal motion during a pipetting operation. The 3D trajectory surface illustrates temporally ordered positional and orientation changes, with color indicating progression through the operation.

Figure 3, Figure 4, Figure 5 and Figure 6 present pose- and motion-based visualizations of a representative pipetting operation, showing changes in position and orientation over time with a consistent temporal ordering across the sequence.

Figure 3 shows pose-based feature extraction from video data. Hand and arm keypoints are used to estimate the orientation of the pipette, and the inclination angle relative to the vertical direction in the camera coordinate system is plotted along the motion trajectory. The angle values vary over time, reflecting changes in pipette orientation during the operation. Hand keypoints are used to support pipette localization and orientation estimation, while the reported angle corresponds to the pipette axis. Temporal progression is indicated using color coding.

Figure 4 presents the geometric reconstruction of pipette orientation in a camera-centered coordinate system. Using pose-derived keypoints, the pipette axis is reconstructed in three dimensions, and the inclination angle relative to the vertical direction is computed for a representative operation.

Figure 5 shows the temporal evolution of pipette motion and orientation. The axial displacement plot illustrates changes in relative position along the horizontal, vertical, and depth directions over time, while the Euler angle curves depict corresponding variations in pipette orientation throughout the operation.

Figure 6 provides a three-dimensional visualization of pose-derived spatiotemporal features for a single pipetting sequence. The trajectory represents coordinated motion of the pipette and hand, with color indicating temporal progression. Variations in spatial position and orientation are observed across the duration of the operation.

### 4.2. Classification Performance

The classification performance results shown in Table 3, were evaluated using seven different classifiers: ID3, AdaBoost, C4.5, Naive Bayes, Bayesian Network, Random Forest, and Support Vector Machine (SVM). Among these classifiers, ID3 achieved the highest performance, with an impressive accuracy rate of 100% in both the control and experimental groups. Naive Bayes followed closely with 93.3% accuracy across both groups. In contrast, Bayesian Network and SVM classifiers showed weaker performance, particularly in recognizing the subtle distinctions between positive and negative operational categories.

**Figure 7:**
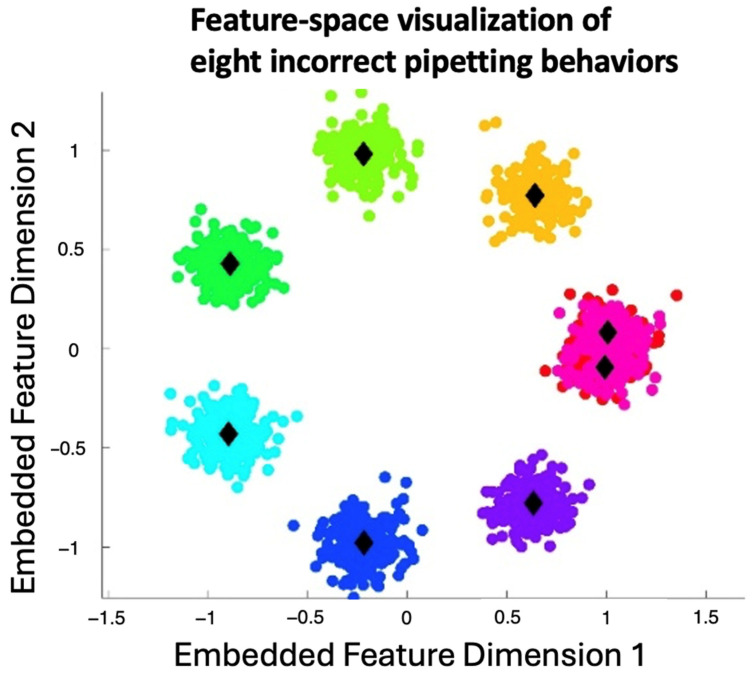
Two-dimensional visualization of sequence-level spatiotemporal feature embeddings for eight incorrect pipetting behaviors. Each point represents one operation sequence, with colors indicating behavior categories and black markers denoting cluster centroids.

The significant variance in classifier performance can be attributed to the nature of the dataset and the task’s characteristics. The input features in this study were discontinuous, presenting a challenge for certain algorithms, which typically excel with continuous data. As such, decision tree-based methods like ID3, which are designed to handle discrete values efficiently, performed the best. This aligns with previous studies in disease classification where ID3 has shown high reliability for tasks involving categorical outcomes. On the other hand, Naive Bayes performed reasonably well due to its probabilistic nature, but its ability to handle such dynamic sequences with temporal dependencies was less robust compared to ID3. Qualitatively, Figure 7 presents a two-dimensional embedding of sequence-level spatiotemporal features for eight incorrect pipetting behavior categories, with cluster centroids in black markers.

### 4.3. Ablation Study Results

Table 4 summarizes the quantitative ablation results under different feature configurations. The static-only and motion-only variants yield lower accuracy and F1-score compared with the full model. The configuration that combines static pose and motion features achieves the highest overall performance across all evaluated metrics.

## 5. Discussions

The results indicate that vision-based sensing combined with deep learning provides a viable approach for monitoring pipetting behaviors in laboratory settings. By integrating pose-based spatial features with motion information and modeling their temporal evolution using a BiLSTM network, the proposed framework captures both postural configuration and execution dynamics associated with pipetting operations. Due to the limited sample size, a formal quantitative ablation study was not conducted; instead, qualitative visual analyses were used to examine the contributions of static pose and motion features.

The visualizations in Figure 5 and Figure 6 illustrate the coordinated movement of the hand and pipette during operation, highlighting consistent positional and orientational changes over time. Figure 7 provides a qualitative view of the distribution of sequence-level spatiotemporal features across eight incorrect pipetting behavior categories. While several categories form relatively compact clusters in the embedded feature space, partial overlap is observed, reflecting similarities in motion patterns and execution order among certain error types. This observation underscores the inherent difficulty of distinguishing closely related operational errors based solely on visual cues and emphasizes the importance of temporal modeling and supervised classification.

The comparatively weaker performance of the Bayesian Network and SVM classifiers may be related to the characteristics of the extracted features, which exhibit discontinuous and time-varying behavior. In contrast, decision tree–based methods such as ID3 appear better suited to handling these feature properties. This finding is consistent with prior observations in application domains involving structured but non-continuous feature representations and suggests that classifier selection plays an important role in laboratory operation analysis tasks. The comparatively strong performance of ID3 in this study may be related to the characteristics of the extracted features, which include discrete thresholds (e.g., angle ranges, depth limits) derived from laboratory operating criteria. Tree-based models are well suited to handling such structured and non-continuous feature representations without requiring assumptions of feature linearity. However, given the limited sample size, this observation should be interpreted with caution, as decision trees are known to be susceptible to overfitting in small datasets. The classifier comparison in this study is intended to provide an empirical reference rather than to claim the general superiority of any specific model. Given the participant-grouped cross-validation setting and the small number of sequences per test fold, perfect fold-level performance may occur and should not be interpreted as evidence of robust generalization.

The ablation results suggest that static pose features and motion features capture complementary aspects of pipetting behavior. While static features encode instantaneous posture and tool configuration, motion features reflect execution dynamics over time. Their combination enables more robust modeling of procedural correctness, particularly for subtle operational errors that cannot be reliably identified using a single feature stream.

While the system performed well with the current dataset, the relatively small sample size and variability in operator behavior may limit the generalization capability of the model. Furthermore, the use of pose-based perception and recurrent temporal modeling has been explored in other application domains [31,32,33], this work focuses on adapting and validating such techniques for fine-grained monitoring of laboratory pipetting operations, where procedural correctness and temporal execution are critical. Future studies will focus on expanding the dataset to include more diverse operational conditions and laboratory environments to improve generalizability, and assessing the system’s generalizability across different experimental environments. Additionally, optimizing classifiers such as Random Forest and SVM with feature engineering could improve the system’s performance for more complex datasets.

Although the proposed framework is designed to operate as a non-contact system without modifying existing laboratory equipment, several practical deployment challenges remain. Variations in lighting conditions, partial occlusions of the hand or pipette, and the presence of multiple operators within the camera field of view may affect perception accuracy in real-world laboratory environments. While the current experiments were conducted under controlled conditions, future work will focus on improving robustness through data augmentation strategies, illumination-invariant feature learning, and multi-view or multi-camera sensing. In addition, extending the framework to handle multi-operator scenarios using multi-person pose tracking and identity association represents an important direction for deployment in shared or high-throughput laboratory spaces.

Extending the framework to multi-site or cross-institutional settings would further improve generalizability but also raises privacy and data governance concerns for video-based behavioral data. Privacy-preserving collaborative learning approaches, such as federated learning combined with differential privacy, offer a promising direction for enabling distributed model training without centralized sharing of raw video data [34]. In addition, secure data governance mechanisms, including blockchain-based access control and auditability frameworks, may support controlled access and traceability when operational videos and derived logs are shared across laboratories or users [35].

In addition to human operation monitoring, the proposed framework may also support future laboratory automation systems. By characterizing fine-grained motion patterns and operational constraints from real human operators, the extracted spatiotemporal features and error definitions could inform quality assessment of robotic pipetting systems and provide guidance for the design and evaluation of automated laboratory instruments.

## 6. Conclusions

This study demonstrates the effectiveness of integrating vision-based sensing and deep learning techniques for automated monitoring of pipetting behaviors in chemical laboratory settings. By combining YOLOv8 for pose estimation with BiLSTM for temporal modeling, the framework accurately classifies correct and incorrect pipetting operations. The results show that both motion and static features are crucial for optimal performance, and the ID3 classifier provided the best results. Despite the promising outcomes, future work should focus on expanding the dataset, optimizing classifiers, and enhancing system generalizability across diverse experimental conditions. This work provides a foundation for improving laboratory safety, training, and procedural adherence through AI-enabled monitoring systems.

## Figures and Tables

**Table 1:** Operational definitions of incorrect pipetting behaviors used in this study.

No.	Incorrect Behavior	Error Category	Behavior Description (Standards/Regulatory Rationale)	Operational Criteria (Automation Detection)
1	Vertical insertion of pipette tip into liquid	Postural Errors	According to laboratory operation standards, the pipette tip should be inserted into the liquid at an inclined angle (typically 15–45°). Vertical insertion increases the risk of contacting the container bottom, causing sample contamination, tip blockage or damage, and bubble formation that degrades volumetric accuracy.	Angle between the pipette tip and the vertical direction is smaller than 15° or larger than 75° (threshold adjustable based on protocol).
2	Excessive or insufficient immersion depth of pipette tip	Tool Handling Errors	The immersion depth should be moderate (typically 2–5 mm, depending on container and liquid properties). Excessive depth may cause liquid adhesion on the outer wall or aspiration of foam, while insufficient depth may introduce air, resulting in inaccurate volume transfer.	Tip immersion depth exceeds the predefined upper limit (e.g., >10 mm) or falls below the lower limit (e.g., <1 mm).
3	Contact between pipette tip and container wall	Tool Handling Errors	The pipette tip should not touch the container wall to prevent cross-contamination, liquid adhesion, volumetric loss, or damage to the tip.	Horizontal distance between the pipette tip and the container inner wall is smaller than a safety threshold (e.g., <0.5 mm).
4	Incomplete liquid dispensing (including residual droplets)	Sequential Errors	Pipetting operations should ensure complete liquid dispensing. In forward pipetting, residual liquid should be expelled by touching the container wall; reverse pipetting should follow protocol-specific steps. Residual liquid leads to inaccurate reagent delivery and reaction bias.	Visible liquid residue remains inside the pipette tip after dispensing (vision-based detection) or measured mass change does not meet the expected value (weight-based sensing).
5	Reuse of the same pipette tip for different reagents or samples	Tool Handling Errors	Pipette tips should be single-use or replaced when switching reagents or samples. Reuse may cause cross-contamination, reagent degradation, and experimental interference.	The same pipette tip is detected contacting different reagent containers or samples without being replaced.
6	Failure to discard or clean pipette tip after use	Tool Handling Errors	After pipetting, disposable tips should be immediately discarded, or reusable pipettes should be cleaned according to protocol. Residual reagents may contaminate subsequent experiments, corrode equipment, or promote microbial growth.	After a pipetting operation, no discard or cleaning action is detected within a predefined time window.
7	Excessively fast liquid dispensing	Motion Patterns Errors	Dispensing speed should be smooth and controlled. Rapid dispensing may generate bubbles, splashing, or residual droplets, adversely affecting volumetric accuracy and reaction homogeneity.	Dispensing rate exceeds a predefined threshold (e.g., >1 mL/s, adjustable based on liquid viscosity).
8	Reading volume before liquid stabilization or without parallax correction	Sequential Errors	Volume should be read only after the liquid surface stabilizes (typically after 3–5 s), with the observer’s eye aligned horizontally with the lowest point of the meniscus to avoid parallax error.	Volume reading occurs before surface oscillation ceases within the predefined time, or the viewing angle deviates beyond the allowable range from the horizontal plane.

**Table 2:** Summary of participants and pipetting trials.

Participant Group	No. of Participants	Trials per Participant	Total Sequences
Novice	6	4	24
Experienced	6	4	24
Total	12	—	48

**Table 3:** Classification performance comparison of seven classifiers on the pipetting behavior dataset. The table summarizes accuracy, precision, recall, and F1-score for each classifier, highlighting the best-performing algorithms and their robustness in identifying correct and incorrect pipetting operations.

	Negative			Positive		
	Precise	Recall	F1score	Precise	Recall	F1score
ID3	100%	100%	100%	100%	100%	100%
AdaBoost	85.71%	80%	82.76%	81.25%	86.67%	83.87%
C45	100%	86.67%	92.86%	88.24%	100%	93.75%
Bayes	0	0	0	50%	100%	66.67%
Naive Bayes	93.33%	93.33%	93.33%	93.33%	93.33%	93.33%
Random forest	100%	80%	88.89%	83.33%	100%	90.91%
Support Vector Machine	61.54%	53.33%	57.14%	58.82%	66.67%	62.5%

**Table 4:** Results of the ablation study under different feature configurations. ✓ indicates that selective features are included; ✗ indicates that selective features are not included.

Variant	Pose Source	Motion	Static	Fusion	Temporal Model/Classification Head	Accuracy	F1-Score (Standard Operations)	F1-Score (Erroneous Operations)	Macro Avg. F1
A1	Ground-truth	✓	✓	Concat	LSTM + FC (Softmax)	98.5	99.0	98.0	98.5
A2	YOLOv8	✗	✓	—	SVM	75.2	80.1	70.3	75.2
A3	YOLOv8	✓	✗	—	SVM	72.8	78.5	67.1	72.8
A4	YOLOv8	✓	✓	Average	LSTM + FC (Softmax)	89.3	90.5	88.1	89.3
A5	YOLOv8	✓	✓	Concat	Random Forest	91.0	92.2	89.8	91.0
Full	YOLOv8	✓	✓	Concat	LSTM + FC (Softmax)	95.7	96.5	94.9	95.7

## Data Availability

The data are available from the corresponding author upon reasonable request.

## Abbreviations

AI	Artificial Intelligence
BiLSTM	Bidirectional Long Short-Term Memory
LSTM	Long Short-Term Memory
YOLO	You Only Look Once
QA	Quality Assurance
ROI	Region of Interest
ID3	Iterative Dichotomiser 3
C4.5	C4.5 Decision Tree Algorithm
SVM	Support Vector Machine
WLD	Weber Local Descriptor
GLP	Good Laboratory Practice
GMP	Good Manufacturing Practice
GB/T	Guobiao Recommended Standard (China National Recommended Standard)
GLP/GMP	Good Laboratory Practice/Good Manufacturing Practice

## Appendix A

**Table A1:** Rule-based operational definitions and threshold values for eight incorrect pipetting behaviors. Thresholds are defined relative to camera calibration and experimental setup and can be adjusted for different laboratory configurations.

No.	Incorrect Behavior	Operational Criterion	Threshold Value	Error Description
1	Vertical insertion of pipette tip into liquid	${Error}_{1}=\left\{\begin{array}{c} 1,\theta <\theta_{min}\;OR\;\theta >\theta_{max}\\ 0,\mathrm{otherwise} \end{array}\right.$	$\theta_{min}=30{}^{\circ}$ $\theta_{max}=80{}^{\circ}$	${Error}_{1}=1$ if the pipette insertion angle θ falls outside the allowable range [$\theta_{min}$, $\theta_{max}$].
2	Excessive or insufficient immersion depth of pipette tip	${Error}_{2}=\left\{\begin{array}{c} 1,d>d_{max}\\ 0,\mathrm{otherwise} \end{array}\right.$	$d_{max}=6\;mm$ (for standard microcentrifuge tubes)	${Error}_{2}=1$ when the pipette tip immersion depth d exceeds the maximum allowable depth $d_{max}$.
3	Contact between pipette tip and container wall	${Error}_{3}=\left\{\begin{array}{c} 1,\delta <\delta_{min}\\ 0,\mathrm{otherwise} \end{array}\right.$	$\delta_{min}=1.5\;mm$	${Error}_{3}=1$ when the distance δ between the pipette tip and the container wall is less than the minimum allowable distance $\delta_{min}$.
4	Incomplete liquid dispensing (including residual droplets)	${Error}_{4}=\left\{\begin{array}{c} 1,V_{residual}>V_{max}\\ 0,\mathrm{otherwise} \end{array}\right.$	$V_{max}=95\%$	${Error}_{4}=1$ when the residual liquid volume $V_{residual}$ after dispensing exceeds the allowable maximum V_max_, indicating incomplete dispensing.
5	Reuse of the same pipette tip for different reagents or samples	${Error}_{5}=\left\{\begin{array}{c} 1,\exists \left(i,j\right),C_{i,j}>C_{max}\\ 0,\mathrm{otherwise} \end{array}\right.$	$C_{max}=0$	${Error}_{5}=1$ when the contamination index $C_{i,j}$ between reagents i and j exceeds the allowable threshold $C_{max}$
6	Failure to discard or clean pipette tip after use	${Error}_{6}=\left\{\begin{array}{c} 1,CleanFlag=0⋀t_{idle}>t_{corrosion}\\ 0,\mathrm{otherwise} \end{array}\right.$	$t_{corrosion}=5\;min$	${Error}_{6}=1$ when the pipette is not marked as cleaned (CleanFlag=0) and the idle time $t_{idle}$ exceeds the allowable threshold $t_{corrosion}$, indicating delayed cleaning.
7	Excessively fast liquid dispensing	${Error}_{7}=\left\{\begin{array}{c} 1,\frac{dv}{dt}>a_{max}\\ 0,\mathrm{otherwise} \end{array}\right.$	$a_{max}$ = 0.5 $V_{target}/s$	${Error}_{7}$ = 1 when the absolute volumetric dispensing rate $\frac{dv}{dt}$ exceeds the maximum allowable rate $a_{max}$
8	Reading volume before liquid stabilization or without parallax correction	${Error}_{8}=\left\{\begin{array}{c} 1,\sigma \left(t\right)>\sigma_{max}⋀t_{wait}<t_{stable}\\ 0,\mathrm{otherwise} \end{array}\right.$	$\sigma_{max}$ = 0.5% $V_{target}/s$ $t_{stable}$ = 2 s	${Error}_{8}$ = 1 when the meniscus instability $\sigma \left(t\right)$ exceeds the allowable limit $\sigma_{max}$ and the waiting time $t_{wait}$ is shorter than the required stabilization time $t_{stable}$.
